# Dynamical dissipative and radiative flow of comparative an irreversibility analysis of micropolar and hybrid nanofluid over a Joule heating inclined channel

**DOI:** 10.1038/s41598-023-31920-1

**Published:** 2023-04-01

**Authors:** S. Suresh Kumar Raju

**Affiliations:** Department of Mathematics and Statistics, College of Science, King Faisal University, Al-Ahsa, 31982 Saudi Arabia

**Keywords:** Mathematics and computing, Nanoscience and technology, Mechanical engineering, Theory and computation

## Abstract

This report scrutinized the influence of radiation and Ohmic heating on the dissipative flow of micropolar and hybrid nanofluid within an inclined length $2h$ channel under convective boundary conditions. Primary flow equations are renewed as the system of NODEs with the assistance of proper similarity conversions. In two circumstances, hybrid fluid flow and micropolar fluid flow, a blend of shooting and Runge–Kutta 4th order strategy, is used to achieve the desired results. The critical consequences of the current study are Larger pressure gradient minimizes the fluid velocity, and a more significant inertia parameter minimizes the rotation profile in the case of Newtonian fluid flow but facilitates the same in the case of hybrid nanofluid flow. It is perceived that the escalation in Brinkmann number causes the amelioration in the fluid temperature, and the radiation parameter mitigates the same. Furthermore, it is discovered that the Grashoff number enhances the Bejan number at the centre of the channel but lessens the same at other areas. Finally, validation is executed to compare the current outcomes with the former results and perceive a good agreement.

## Introduction

Hydrous or unsteady globes might also be electrically conductive and capable of withstanding core fluxes commencing electromagnetic stimulation. Instances of this occurrence are occasionally named induction boiler or Joule heating. These constituents of ohmic heating are offered in various manufacturing, industrial and cosmological sceneries. By viewing this, Makinde and Gbolagade^1^ investigated entropy generation in a laminar viscous fluid flow via an inclined passage. They discovered that fluid friction's irreversibility dominated heat transfer's irreversibility on the channel centerline. Guimaraes and Menon^2^ conducted a heat transmission investigation of mixed convective fluid within an inclined channel (rectangular) with the help of the finite element technique. Dar and Elangovan^3^ inspected the impact of a magnetic field on the peristaltic flow through an inclined channel (asymmetric) and acknowledged that the magnetic field lessens the fluid velocity. Shahri and Sarhaddi^4^ emphasized that the main reason for entropy generation is the heat conduction of nanofluid (water–Cu) in their examination of MHD fluid flow within an inclined channel. By assuming a low Reynolds number and considering an inclined channel, Javed et al.^5^ scrutinized the peristaltic flow with the Hartmann number. They concluded that the Hartmann number escalates the trapped bolus size. Hayat et al.^6^ analyzed the peristaltic transport of the pseudoplastic fluid flow in the same parameter with heat source and Joule heating. Reynolds number ameliorates the fluid temperature is one of the findings of this study. Tlau and Ontela^7^ considered convective conditions and elucidated the mixed convective flow of $H_{2} O + Cu$ a tended channel occupied with a permeable medium. They observed the enhancement in the fluid velocity with a greater inclination angle. With the assumption of the same geometry, Adesanya et al.^8^ and Singh et al.^9^ proposed a model for different fluid flows to discuss the irreversibility analysis. They discovered that there is a reduction in the entropy generation rate with a couple of stress parameters. Sabu et al.^10^ used a correlation coefficient to examine the features of engineering parameters in an unsteady MHD nanofluid flow with the heat source. They detected that the Soret number is negatively affiliated with the Sherwood number. Several researchers^11–14^ recently examined different fluid flows (including hybrid nanofluid) via similar geometry and highlighted that inclined geometry control the flow and heat transfer process.

Improvement in the transfer of heat across fluid movement has made authorities in thermal manufacturing encirclement the efficiency of a combination of solid nanoparticles called a Hybrid nanofluid. The previously revealed improvement is based on the nature of the base fluid and nanoparticles. Solid particle concentration and thermal properties on the proportion of mass to density and viscidness are precisely the physical possessions. Nevertheless, thermal conductivity and specific heat capacity at different intensities of concentration of nano-solid particles, nanoparticles' size, and temperature are some of the thermal possessions. Considering this, Gholinia et al.^15^ illustrated the MHD flow of a nanofluid (Ethylene glycol + Silver + Copper) by a circular cylinder with injection/suction. They concluded that the silver nanoparticles are better than copper when a higher temperature is required. Nadeem et al.^16^ numerically investigated a nanofluid (Water + SWCNT) flow by a curled sheet with a magnetic field. They observed that the volume fraction of nanoparticles ameliorates the fluid temperature. Sowmya et al.^17^ assumed longitudinal fin as geometry and examined the convective flow of a nanofluid (alloys of titanium and aluminium) with radiation. Dogonchi et al.^18^ inspected the radiative flow of $Cu + H_{2} O$ fluid with a heat source and two reactions (heterogeneous–homogeneous) by a flat plate. They found a positive association between the Nusselt number and the magnetic field parameter. Newly, Anuar et al.^19^ and Waqas et al.^20^ assumed distinct geometries and scrutinized different water-based nanofluid flows under various conditions. Jamshed and Aziz^21^ did an irreversibility analysis on the Casson HNF $\left( {TiO_{2} - CuO/EG} \right)$ flow by an elongating surface with the CCHF model. They found that the Brinkman number escalates the entropy generation. Salman et al.^22^ considered FFS and BFS and reviewed various hybrid nanofluid flows. They opined that HNFs are the best alternates compared to mono NFs when better thermal features are required. Abbas et al.^23^ assumed a thin needle and inspected the forced convective flow of an HNF (Water + SWCNT + MWCNT) with variable thermal conductivity. Anuar et al.^24^ and Waini et al.^25^ delivered a stability study for the radiative HNF $\left( {Cu - Al_{2} O_{3} /Water} \right)$ flow by a revolving shrinking/elongating sheet. Based on that, they categorized the solutions as stable and unstable. Recently, various researchers^26–37^ considered different geometries as well as the combination of solid nanoparticles and generated intermediate kinds of conductivity properties. This helps us to highlight the intermediatory processes.

Once the careful insight of the earlier stated inscription, we propose to discourse the importance of the radiation and Ohmic heating on the dissipative flow of micropolar and combination of solid nanoparticles (Propylene glycol − Water mixture + Paraffin Wax + Sand) by a tended channel. Results are offered in two examples, i.e., micropolar fluid and HNF. Further, irreversibility analysis is performed for the fluid flow against various pertinent parameters. The outcome of such investigation could be useful to identify the performances of nanoparticles and their effectiveness in microchannels filled with low conductivity properties. The following investigation was modelled to provide the answers to the following related research queries.

1. When non-linear energy and mass flux due to concentration and thermal gradient are negligible, what is the importance of the growing radius of solid nanoparticles on the entropy generation and Bejan numbers?
2. At different intensities of energy and momentum fluxes due to gradients in momentum and thermal, respectively, how do PEG-based Magnesium oxide $\left( {MgO} \right)$ and Zirconium oxide $\,(ZrO_{2} )$ nanoparticles influence the transport phenomena of an inclined channel?
3. When irreversibility takes place, what variations are availed for micro-polar and hybrid nanofluid?

## Mathematical formulation

In this model, we have considered a non-transient steady laminar incompressible radiative hybrid and micropolar nano fluid flow in a microchannel of width $h$. Magnesium oxide $\left( {MgO} \right)$ and Zirconium oxide $\,(ZrO_{2} )$ nanoparticles are considered with a base fluid polyethene glycol (PEG). Values of the thermo-physical attributes are presented in Table 1.

**Figure 1:**
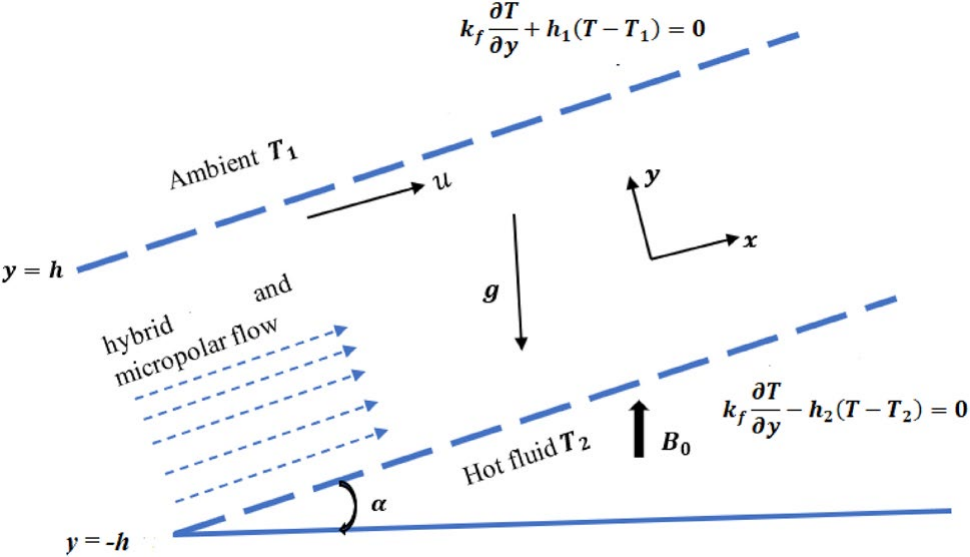
Representation of the flow.

The channel, which has an inclination angle $\alpha$, is formed by two plates (upper and lower), separated by a distance $2h$ and positioned at $y = h$ and $y = - h$ correspondingly. The $x$ axis is located in the centre of the channel, which indicates the direction of the flow. Let the bottom plate of the microchannel exchange the hot fluid temperature $T_{2}$ via convection, whereas the top plate is in contact temperature with the ambient fluid temperature $T_{1}$. Magnetic field (MHD) of potential strength $B_{0}$ is imposed in the $y$ direction, to record its influence on flow and heat transformation. The above stated assumptions are depicted in Fig. 1.

Under the above-mentioned assumptions, the flow governing equations following (Srinivasacharya and Bindu^38^, Xiangcheng and Shiyuan Li^39^ and Roja et al.^40^), are

1 $$\upsilon = \upsilon_{0}$$

2 $$\mu_{hnf} \left( {1 + \kappa } \right)\frac{{\partial^{2} u}}{{\partial y^{2} }} - \rho_{hnf} \upsilon_{0} \frac{\partial u}{{\partial y}} + \kappa \frac{\partial N}{{\partial y}} + \left( {\rho \beta } \right)_{hnf} g\left( {T - T_{1} } \right)\sin \left( \alpha \right) - \sigma B_{0}^{2} u - \frac{\partial p}{{\partial x}} = 0$$

3 $$\gamma \frac{{\partial^{2} N}}{{\partial y^{2} }} - \rho_{hnf} j\upsilon_{0} \frac{\partial N}{{\partial y}} - 2\kappa N - \kappa \frac{\partial u}{{\partial y}} = 0$$

4 $$\begin{gathered} \left( {k_{hnf} + \frac{{16\sigma *T_{1}^{3} }}{3k*}} \right)\frac{{\partial^{2} T}}{{\partial y^{2} }} - \left( {\rho C_{p} } \right)_{hnf} \upsilon_{0} \frac{\partial T}{{\partial y}} + \left( \begin{gathered} \mu_{hnf} \hfill \\ + \kappa \hfill \\ \end{gathered} \right)\left( {\frac{\partial u}{{\partial y}}} \right)^{2} + 2\kappa \left( {N^{2} + N\frac{\partial u}{{\partial y}}} \right) \hfill \\ + \left( \begin{gathered} Q_{T}^{*} (T - T_{1} ) \hfill \\ + \sigma B_{0}^{2} u \hfill \\ \end{gathered} \right) + \gamma \left( {\frac{\partial N}{{\partial y}}} \right)^{2} = 0 \hfill \\ \end{gathered}$$

with the conditions (Srinivasacharya and Bindu^38^)

5 $$at\;y = - h:\;u = 0,\;N = - n\frac{\partial u}{{\partial y}},\;k_{f} \frac{\partial T}{{\partial y}}\; - h_{2} \left( {T - T_{2} } \right) = 0$$

$$at\;y = h:\;u = 0,\;N = 0,\;k_{f} \frac{\partial T}{{\partial y}} + h_{1} \left( {T - T_{1} } \right) = 0$$

**Table 1:** Numerical values of thermophysical attributes convoluted in HNF (Hossainy and Eid^41^).

S.no		Polyethylene glycol–water mixture $\left( f \right)$	$ZrO_{2}$ $\left( {s_{1} } \right)$	$MgO$ $\left( {s_{2} } \right)$
1	$\rho \left( {{\text{Kg}}/{\text{m}}^{3} } \right)$	1110	5680	3560
2	$k\left( {{\text{Wm}}^{ - 1} {\text{K}}^{ - 1} } \right)$	0.3712	1.7	45
3	$C_{p} \left( {{\text{JKg}}^{ - 1} {\text{K}}^{ - 1} } \right)$	3354	502	955

and 

**Table:** 

$\rho_{hnf} = \left( {1 - \phi_{2} } \right)\left[ {\left( {1 - \phi_{1} } \right)\rho_{f} + \phi_{1} \rho_{{s_{1} }} } \right] + \phi_{2} \rho_{{s_{2} }}$	$\frac{{\sigma_{nf} }}{{\sigma_{f} }} = 1/\left( {\frac{{\sigma_{{s_{1} }} + 2\sigma_{f} + \phi_{1} \left( {\sigma_{f} - \sigma_{{s_{1} }} } \right)}}{{\sigma_{{s_{1} }} + 2\sigma_{f} - 2\phi_{1} \left( {\sigma_{f} - \sigma_{{s_{1} }} } \right)}}} \right)$
$\mu_{hnf} = \frac{{\mu_{f} }}{{\left( {1 - \phi_{1} } \right)^{2.5} \left( {1 - \phi_{2} } \right)^{2.5} }}$	$k_{hnf} = \frac{{k_{{s_{2} }} + 2k_{nf} - 2\phi_{2} \left( {k_{nf} - k_{{s_{2} }} } \right)}}{{k_{{s_{2} }} + 2k_{nf} + \phi_{2} \left( {k_{nf} - k_{{s_{2} }} } \right)}} \times k_{nf}$
$\sigma_{hnf} = \frac{{\sigma_{{s_{2} }} + 2\sigma_{nf} - 2\phi_{2} \left( {\sigma_{nf} - \sigma_{{s_{2} }} } \right)}}{{\sigma_{{s_{2} }} + 2\sigma_{nf} + \phi_{2} \left( {\sigma_{nf} - \sigma_{{s_{2} }} } \right)}} \times \sigma_{nf}$	$k_{nf} = \frac{{k_{{s_{1} }} + 2k_{f} - 2\phi_{1} \left( {k_{f} - k_{{s_{1} }} } \right)}}{{k_{{s_{1} }} + 2k_{f} + \phi_{1} \left( {k_{f} - k_{{s_{1} }} } \right)}} \times k_{f}$
$$\left( {\rho C_{p} } \right)_{hnf} = \left( {1 - \phi_{2} } \right)\left[ {\left( {1 - \phi_{1} } \right)\left( {\rho C_{p} } \right)_{f} + \phi_{1} \left( {\rho C_{p} } \right)_{{s_{1} }} } \right] + \phi_{2} \left( {\rho C_{p} } \right)_{{s_{2} }}$$	

where $N$-micro rotation, $K = \,\frac{\kappa }{{\mu_{f} }}$—micro polar parameter, $\kappa$—vortex viscosity,$\rho$ the density, $\mu$ the dynamic viscosity, $j$ the gyration parameter, $g$ is the gravitation, $\beta$ the thermal expansion coefficient, $q_{r}$ the radiative heat flux, $\sigma$ the electrical conductivity, $\gamma$ the spin gradient viscosity and $\gamma = \left( {\mu_{nf} + \frac{\kappa }{2}} \right)j = \mu_{f} \left( {\frac{{\mu_{nf} }}{{\mu_{f} }} + \frac{K}{2}} \right)j$, and $h_{1} ,h_{2}$—the convective heat transfer coefficient for the individual plate. $n$ Constant and $0 \le n \le 1$ ($n = 0$ represents strong concentration, $n = 0.5$ anti-symmetric part of the stress tensor which represents weak concentration, $n = 1$ is considered while modelling flow problem related to turbulent boundary layer). $\phi_{1}$ and $\phi_{2}$ the volume fraction of $MgO$ and $\,ZrO_{2}$ nanoparticles, subscripts $hnf$ specifies hybrid nanofluid (two nanomaterials + one base fluid), $nf$ specifies nano liquid (one nanomaterial + base liquid), $s_{1}$ and $s_{2}$ specifies solid particles (1-*MgO*, 2-*ZrO*_2_) and $f$ requires base liquid (-polyethylene glycol (PEG)), $k*$ mean absorption coefficient and $\sigma *$ Stefan-Boltzmann constant.

Employing the subsequent non-dimensional variables (Roja et al.^40^)

6 $$\zeta = \frac{y}{h},\,\,\,u = U_{0} f\left( \zeta \right)\,,N = \frac{{U_{0} }}{h}g\left( \zeta \right),\,\,\,T = T_{1} + \left( {T_{2} - T_{1} } \right)\theta \left( \zeta \right)$$

in Eqs. (2)–(5), following the non-linear system of ODEs and their associated boundary conditions are obtained.

7 $$\left( {1 + K} \right)\frac{{d^{2} f}}{{d\zeta^{2} }} = A_{2} \left[ {RA_{1} \frac{df}{{d\zeta }} - K\frac{dg}{{d\zeta }} - \frac{Gr}{{\text{Re}}}A_{3} \theta \sin \alpha + Mf + A} \right]$$

8 $$\frac{{d^{2} g}}{{d\zeta^{2} }} = \left( {\frac{{2A_{2} }}{2 + K}} \right)\left( {\frac{1}{{a_{j} }}} \right)\left[ {A_{1} Ra_{j} \frac{dg}{{d\zeta }} + 2Kg - K\frac{df}{{d\zeta }}} \right]$$

9 $$\frac{{d^{2} \theta }}{{d\zeta^{2} }} = \left( {\frac{1}{{A_{4} A_{41} + \frac{4}{3}Ra}}} \right)\left[ {A_{5} \Pr \frac{d\theta }{{d\zeta }} - Br\left( \begin{gathered} \left( {\frac{1}{{A_{2} }} + K} \right)\left( {\frac{df}{{d\zeta }}} \right)^{2} + 2K\left( {\left( {g\left( \zeta \right)} \right)^{2} + g\left( \zeta \right)\frac{df}{{d\zeta }}} \right) \hfill \\ + M\left( {f\left( \zeta \right)} \right)^{2} + \left( {\frac{2 + K}{{2A_{2} }}} \right)a_{j} \left( {\frac{dg}{{d\zeta }}} \right)^{2} \hfill \\ \end{gathered} \right) + Q_{t} \theta } \right]$$

10 $$at\;\zeta = - 1:\;f = 0,\;g = - n\frac{{d^{2} f}}{{d\zeta^{2} }},\;\frac{d\theta }{{d\zeta }} - Bi_{2} \left( {\theta - 1} \right) = 0$$

$$at\,\,\zeta = 1:\,\,\,\,\,f = 0,\,\,g = 0,\,\,\,\,\frac{d\theta }{{d\zeta }} + Bi_{1} \left( \theta \right) = 0$$.

In the above equations,

**Table:** 

$A_{1} = \left( {1 - \phi_{2} } \right)\left[ {\left( {1 - \phi_{1} } \right) + \phi_{1} \frac{{\rho_{{s_{1} }} }}{{\rho_{f} }}} \right] + \phi_{2} \frac{{\rho_{{s_{2} }} }}{{\rho_{f} }}$	$A_{4} = \frac{{k_{{s_{2} }} + 2A_{41} k_{f} - 2\phi_{2} \left( {A_{41} k_{f} - k_{{s_{2} }} } \right)}}{{k_{{s_{2} }} + 2A_{41} k_{f} + \phi_{2} \left( {A_{41} k_{f} - k_{{s_{2} }} } \right)}}$
$A_{2} = \left( {1 - \phi_{1} } \right)^{2.5} \left( {1 - \phi_{2} } \right)^{2.5}$	$A_{41} = 1/\left( {\frac{{k_{{s_{1} }} + 2k_{f} + \phi_{1} \left( {k_{f} - k_{{s_{1} }} } \right)}}{{k_{{s_{1} }} + 2k_{f} - 2\phi_{1} \left( {k_{f} - k_{{s_{1} }} } \right)}}} \right)$
$A_{3} = \left( {1 - \phi_{2} } \right)\left[ {\left( {1 - \phi_{1} } \right) + \phi_{1} \frac{{\left( {\rho \beta } \right)_{{s_{1} }} }}{{\left( {\rho \beta } \right)_{f} }}} \right] + \phi_{2} \frac{{\left( {\rho \beta } \right)_{{s_{2} }} }}{{\left( {\rho \beta } \right)_{f} }}$	$A_{5} = \left( {1 - \phi_{2} } \right)\left[ {\left( {1 - \phi_{1} } \right) + \phi_{1} \frac{{\left( {\rho C_{p} } \right)_{{s_{1} }} }}{{\left( {\rho C_{p} } \right)_{f} }}} \right] + \phi_{2} \frac{{\left( {\rho C_{p} } \right)_{{s_{2} }} }}{{\left( {\rho C_{p} } \right)_{f} }}$

$R = \frac{{\rho_{f} \upsilon_{0} h}}{{\mu_{f} }}$ the suction/injection, $\Pr = \frac{{\mu_{f} \left( {C_{p} } \right)_{f} }}{{k_{f} }}$ the Prandtl number, $Gr = \frac{{\rho_{f}^{2} g\beta_{f} \left( {T_{2} - T_{1} } \right)h^{3} }}{{\mu_{f}^{2} }}$ the Grashof number, $Br = \frac{{\mu_{f} U_{0}^{2} }}{{k_{f} \left( {T_{2} - T_{1} } \right)}}$ the Brinkman number, $A = \frac{{h^{2} }}{{\mu_{f} U_{0} }}\frac{dp}{{dx}}$ the constant pressure gradient, $Q_{t} = \frac{{Q_{T}^{*} a^{2} }}{{k_{f} }}$ the Fourier heat source or sink, $M = \frac{{\sigma B_{0}^{2} h^{2} }}{{\mu_{f} }}$ the magnetic number, ${\text{Re}} = \frac{{\rho_{f} U_{0} h}}{{\mu_{f} }}$ the Reynolds number, $\,Bi_{i} = - \frac{{hh_{i} }}{{k_{f} }}\,\,for\,i = 1,2$ the Biot number, $a_{j} = \frac{j}{{h^{2} }}$ the micro-inertia parameter, $Ra = \frac{{4\sigma *T_{1}^{3} }}{{k_{f} k*}}$ the radiation parameter.

### Entropy optimization

The volumetric rate of entropy optimization is given (Srinivasacharya and Bindu^38^, Roja et al.^40^) as

11 $$S_{G} = \frac{{k_{f} }}{{T_{1}^{2} }}\left[ {\frac{{k_{hnf} }}{{k_{f} }} + Ra} \right]\left( {\frac{{\partial^{2} T}}{{\partial y^{2} }}} \right) + \frac{{\mu_{hnf} }}{{T_{1} }}\left( \begin{gathered} 1 \hfill \\ + \kappa \hfill \\ \end{gathered} \right)\left( {\frac{\partial u}{{\partial y}}} \right)^{2} + \frac{{2K\mu_{f} }}{{T_{1} }}\left[ \begin{gathered} N^{2} \hfill \\ + N\frac{du}{{dy}} \hfill \\ \end{gathered} \right]\, + \frac{\gamma }{{T_{1} }}\left( {\frac{\partial u}{{\partial y}}} \right)^{2} + Q_{t} \left( {T - T_{1} } \right) + \frac{{\sigma B_{0}^{2} u^{2} }}{{T_{1} }}.$$

With the assistance of (6), Eq. (11) can be revised as

$$Ns = \left[ {\frac{{k_{hnf} }}{{k_{f} }} + Ra} \right]\left( {\frac{{d^{2} \theta }}{{d\zeta^{2} }}} \right) + Q_{t} \theta + \frac{Br}{{T_{p} }}\left( \begin{gathered} \frac{1}{{A_{2} }}\left( {1 + K} \right)\left( {\frac{df}{{d\zeta }}} \right)^{2} + 2K\left( {\left( {g\left( \zeta \right)} \right)^{2} + g\left( \zeta \right)\frac{df}{{d\zeta }}} \right) \hfill \\ + M\left( {f\left( \zeta \right)} \right)^{2} + \left( {\frac{2 + K}{{2A_{2} }}} \right)a_{j} \left( {\frac{dg}{{d\zeta }}} \right)^{2} \hfill \\ \end{gathered} \right)$$

where $N_{s} \, = \,\frac{{h^{2} T_{1}^{2} }}{{k_{f} \left( {\Delta T} \right)^{2} }}S_{G}$ is the dimensionless entropy and $T_{p} = \frac{\Delta T}{{T_{1} }}$.

The Bejan number is given by,

$$Be = \frac{{{\text{Irreversibility}}\;{\text{as}}\;{\text{a}}\;{\text{outcome}}\;{\text{of}}\;{\text{mass}}\;{\text{and}}\;{\text{heat}}\;{\text{transmission}}}}{{{\text{Total}}\;{\text{irreversibility}}}}.$$

That is,

$$Be = \frac{{\left[ {\frac{{k_{hnf} }}{{k_{f} }}} \right]\left( {\frac{{d^{2} \theta }}{{d\zeta^{2} }}} \right)}}{{\left[ {\frac{{k_{hnf} }}{{k_{f} }}} \right]\left( {\frac{{d^{2} \theta }}{{d\zeta^{2} }}} \right) + \frac{Br}{{T_{p} }}\left( \begin{gathered} \frac{1}{{A_{2} }}\left( {1 + K} \right)\left( {\frac{df}{{d\zeta }}} \right)^{2} + 2K\left( {\left( {g\left( \zeta \right)} \right)^{2} + g\left( \zeta \right)\frac{df}{{d\zeta }}} \right) \hfill \\ + M\left( {f\left( \zeta \right)} \right)^{2} + \left( {\frac{2 + K}{{2A_{2} }}} \right)a_{j} \left( {\frac{dg}{{d\zeta }}} \right)^{2} \hfill \\ \end{gathered} \right)}}.$$

## Results and discussion

To solve the transmuted equations, a blend of shooting and Runge–Kutta 4th order strategies is used. In this study, results are provided in two cases, hybrid nanofluid and micropolar fluid.

### Velocity and microrotation profiles

**Figure 2:**
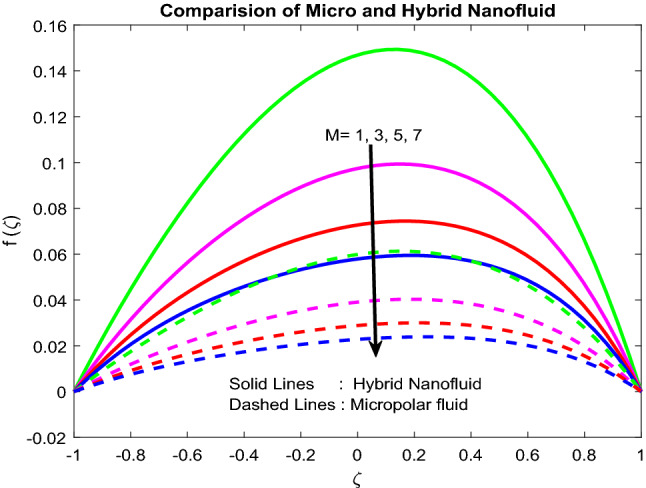
Impact of $$Bi_{2}$$.

**Figure 3:**
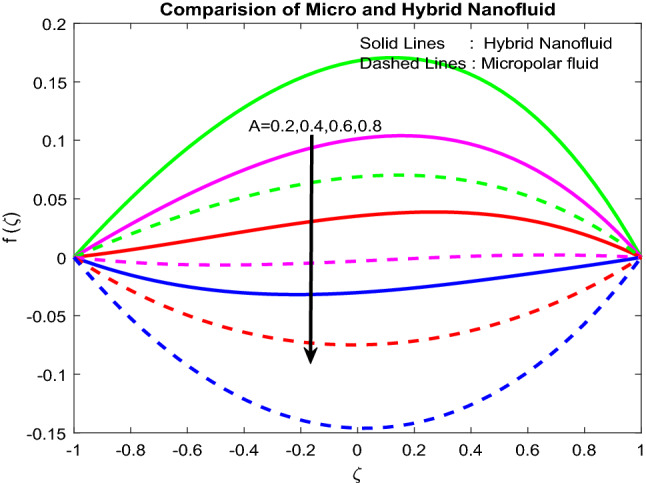
Impact of $$A$$.

**Figure 4:**
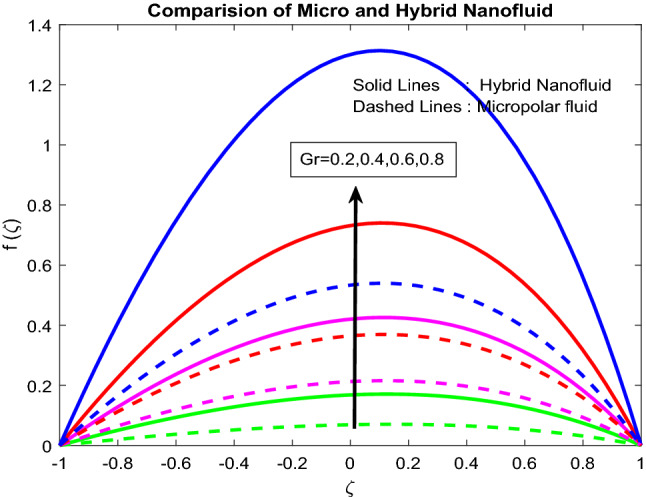
Impact of $$Gr$$.

**Figure 5:**
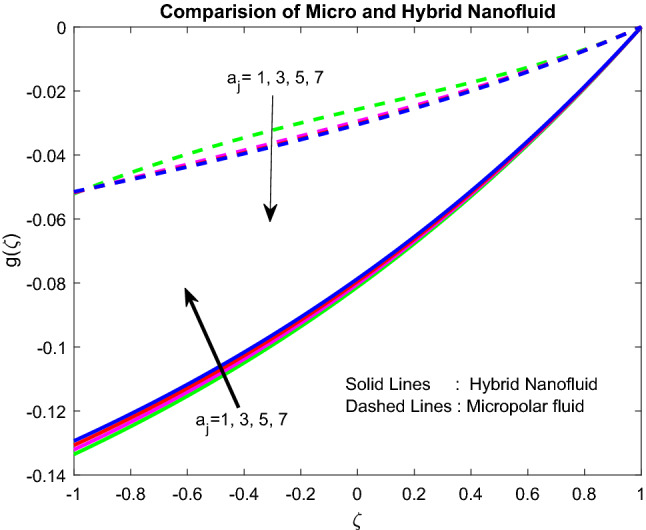
Impact of $${\text{a}}_{j}$$.

The fluid motion is pretentious by a magnetic field. The constituent part of fluid erection, a chain, turns in the direction of the applied attractive field. For the period of this time, the solid particles collide with each other, forming a barrier to the fluid flow. As an outcome, fluid momentum minimizes owing to the upsurge in the viscosity of the liquid (Fig. 2). From Fig. 3, and it is detected that the more significant pressure gradient minimizes the fluid velocity. Physically as expected, the pressure gradient generates higher forces opposing the flow direction, and this causes depreciation in the velocity field. Generally, with the rise in Grashoff number, viscous forces reduce. As a consequence, velocity upsurges (Fig. 4). Figure 5 explicates the fact that the larger inertia parameter minimizes the rotation profile in the case of Newtonian fluid flow and ameliorates the same in the case of hybrid nanofluid flow. Note that the increment in the inertia parameter minimizes the rotation of the particles.

### Temperature profiles

**Figure 6:**
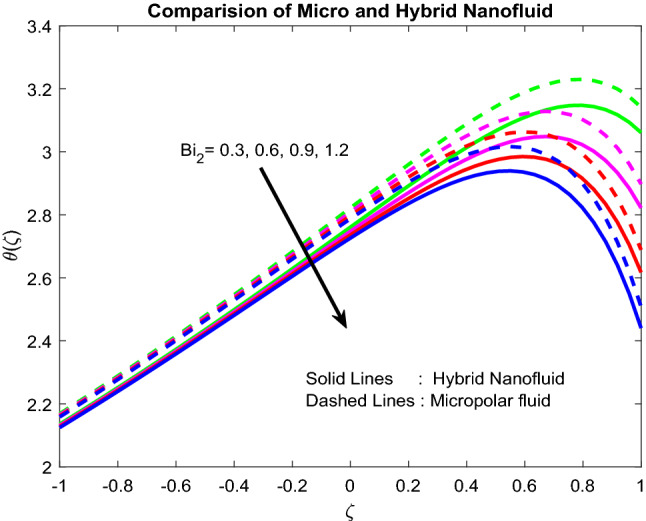
Impact of $$Bi_{2}$$.

**Figure 7:**
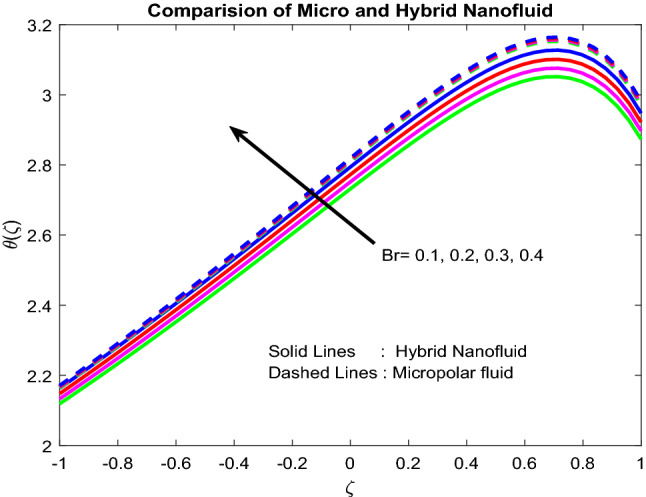
Impact of $$Br$$.

**Figure 8:**
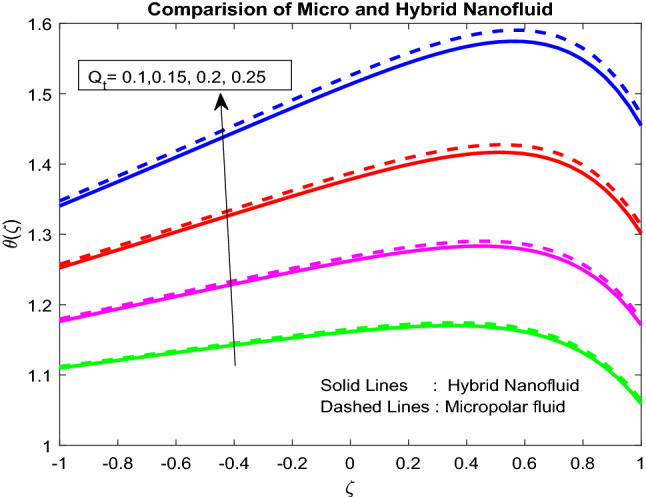
Impact of $$Q_{t}$$.

**Figure 9:**
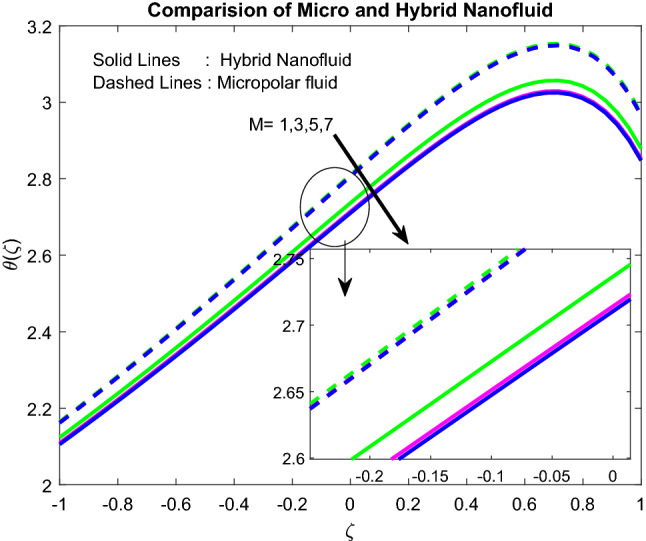
Impact of $$M$$.

**Figure 10:**
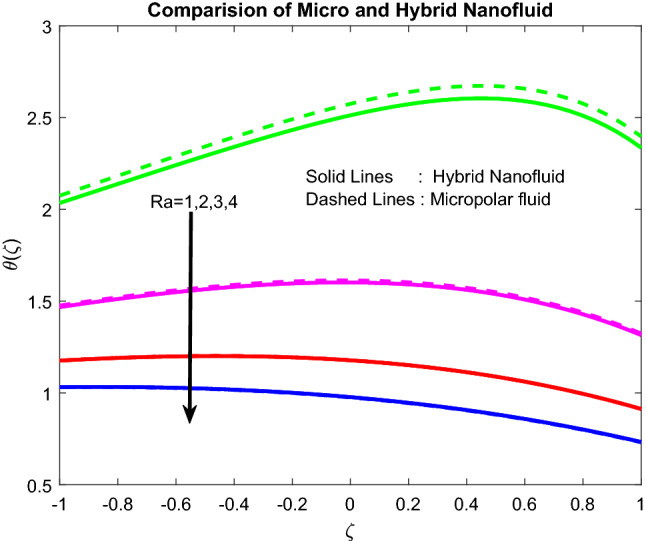
Impact of $$Ra$$.

Figure 6 revealed the fact that the Biot number near the lower channel minimizes the fluid temperature. Typically, with the rise in the Biot number, there is an escalation in the temperature gradient in a positive manner near the lower channel. So, temperature decreases. The higher the value of $Br$, the slower the conduction of heat delivered by viscous dissipation and, subsequently, the bigger the temperature rise (Fig. 7). Figure 8 uncovered that the heat source enriches the fluid temperature. Usually, a higher heat source affects the production of extra hotness inside the liquid and, in order, assists to enhances the thermal boundary. Magnetic field and radiation parameters mitigate the fluid temperature (Figs. 9, 10). Usually, as raising of values, these parameters generate high heat energy and particle interaction. Whereas the inclined nature of the channel sometimes makes to opposes the general physical behaviour, due to this reason, it was reduced.

### Entropy generation and bejan number profiles

**Figure 11:**
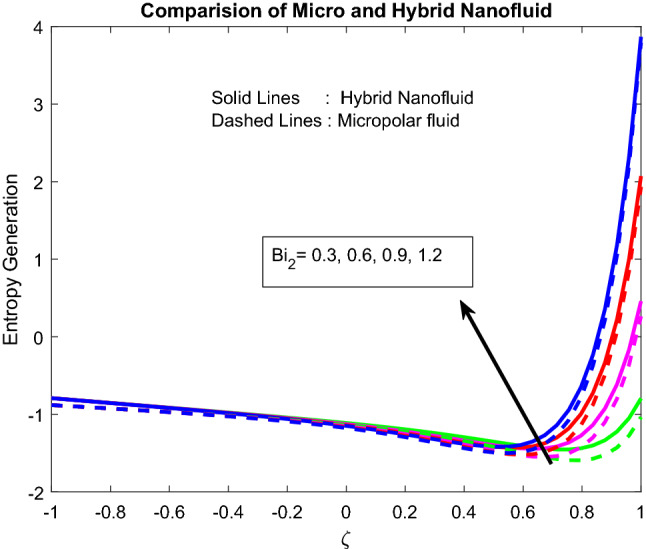
Impact of $$Bi_{2}$$.

**Figure 12:**
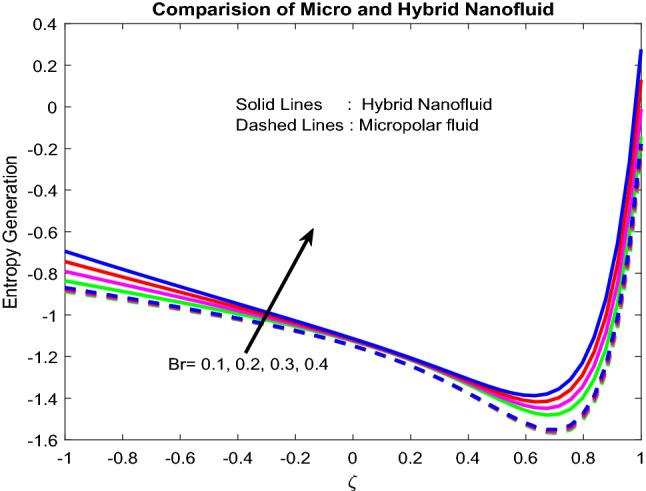
Impact of $$Br$$.

**Figure 13:**
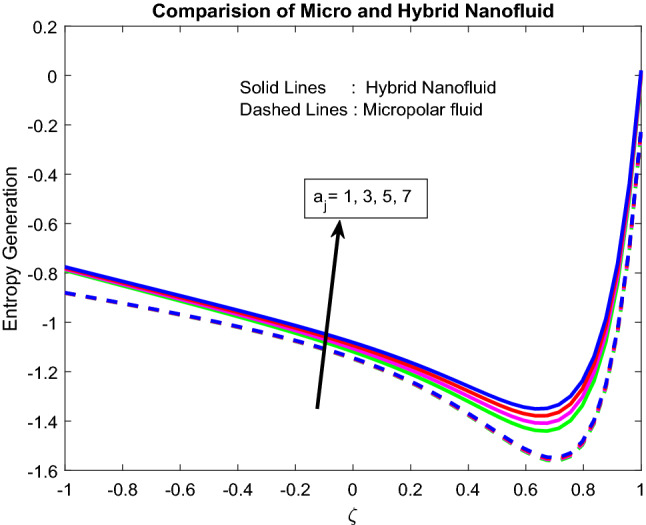
Impact of $${\text{a}}_{j}$$.

**Figure 14:**
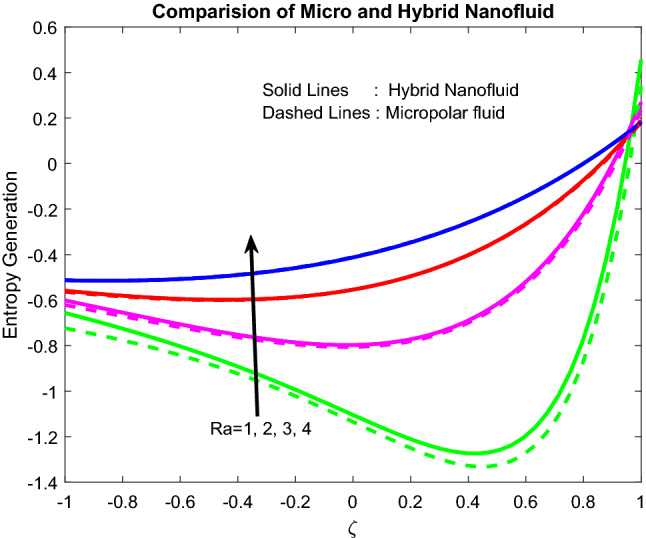
Impact of $$Ra$$.

**Figure 15:**
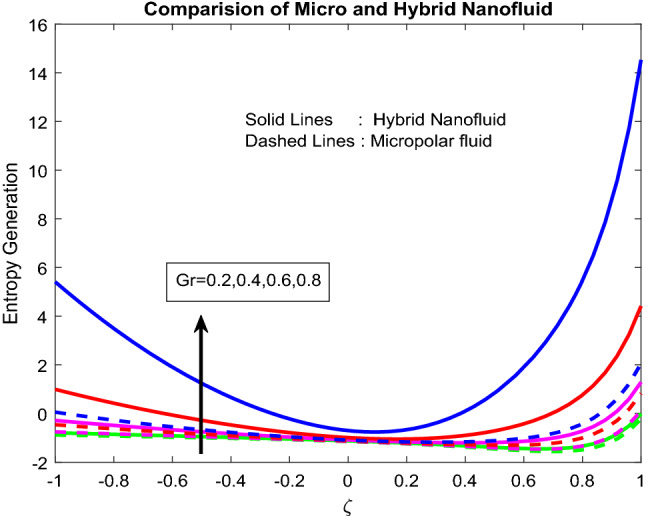
Impact of $$Gr$$.

Figures 11, 12, 13, 14, 15 exhibit the impact of distinct parameters on the entropy generation profile. It is detected that $Bi_{2}$,$Br$,${\text{a}}_{j}$, $Ra$ and $Gr$ are escalating the entropy generation. Note that viscous forces exert friction in fluid flow, which is the primary cause of entropy generation. Bejan number is the proportion of irreversibility because of heat transfer to entire irreversibility because of fluid friction and heat transfer and fluid friction. As raising $Bi_{2}$ improves the convective nature in the help, this helps to encourage the entropy generation, which is displayed in Fig. 11. As micro-inertia impact on entropy generation is shown in Fig. 13 and it is found that improved with ${\text{a}}_{j}$. Since micro inertia is proportional to the entropy generation. A quite similar performance was observed as the raising of Brickman number in Fig. 12. The Fig. 14 displays the thermal radiation on entropy generation. Thermal radiation creates a higher energy to move heat molecules faster, and due to this reason, observed enhancement. As the Grashof number establishes higher pressure, this help to improve interfacial particle transfer, which causes to grow the entropy generation, which is displayed in Fig. 15.

**Figure 16:**
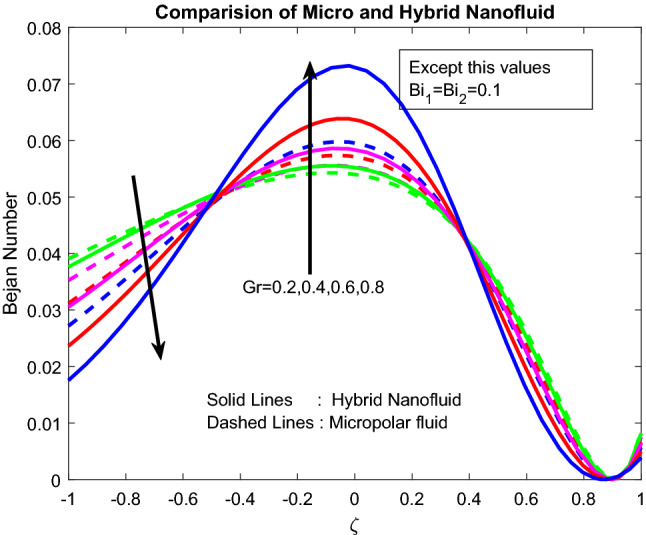
Impact of $$Gr$$.

**Figure 17:**
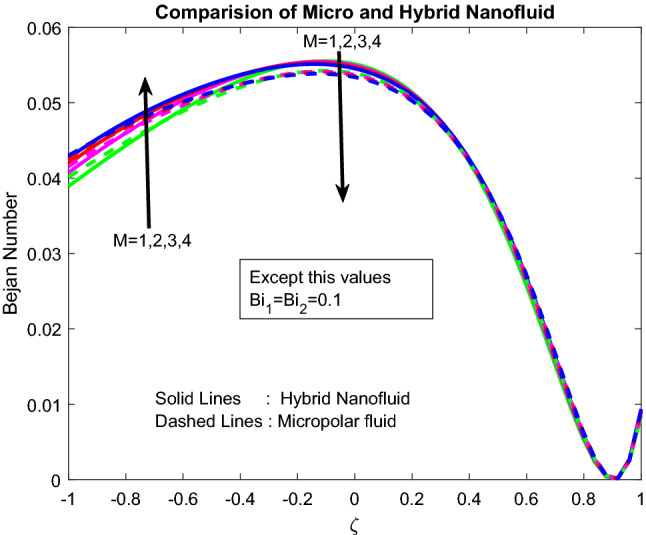
Impact of $$M$$.

**Figure 18:**
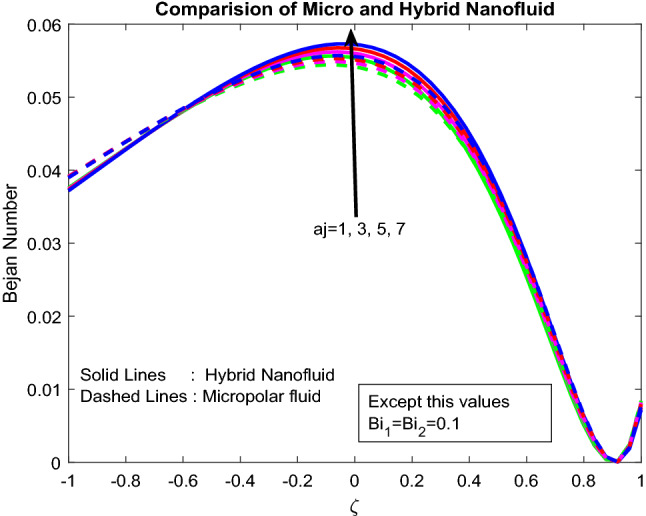
Impact of $${\text{a}}_{j}$$.

Figures 16, 17, 18 explicated the impact of $Gr$, $M$ and ${\text{a}}_{j}$ on Bejan number. It was discovered that $Gr$ it ameliorates the *Be* Bejan number at the midpoint of the channel and minimizes the same at other areas (Fig. 16). This may be owing to the domination of heat transmission irreversibility associated to total irreversibility at the centre of the channel. Parameters $M$ and ${\text{a}}_{j}$ are showing mixed behaviour on Bejan number (Figs. 17, 18). As the well-known fact is, the Bejan number is inversely proportional to the Parameters $M$ and ${\text{a}}_{j}$. But interestingly, the inclined, irreversibility, and convective nature of the flow makes the flow performance in chaotic nature.

### Validation

The current solutions are validated with the existing solution under limited case ($\phi_{1} = \phi_{2} = Gr = M = Q_{t} = n = Ra = R = 0,A = {\text{Re}} = 1,aj = 0$) with the Makinde and Eegunjobi^42^ is shown in Table 2 and found the excellent agreement with those solutions, this help to move further.

**Table 2:** Validation of the current solutions with existing literature when $\phi_{1} = \phi_{2} = Gr = M = Q_{t} = n = Ra = R = 0,A = {\text{Re}} = 1,aj = 0.$

$\zeta$	Makinde and Eegunjobi^42^	Present solutions
0	0	0
0.4	0.1138	0.1138
0.6	0.1215	0.1215
0.8	0.0868	0.0868
1	0	0

## Conclusion

Hydrous or unsteady globes might also be electrically conductive and capable of withstanding core fluxes commencing electromagnetic stimulation. Instances of this occurrence are occasionally named induction boiler or Joule heating. In this report, we aim to scrutinize the influence of Ohmic heating on the radiative and dissipative flow of the micropolar flow of hybrid nanofluid within an inclined channel of length $2h$ under convective boundary conditions. Primary equations of the flow are renewed as the system of ODEs with the aid of proper similarity transmutations. The blend of shooting and Runge–Kutta 4th order approaches are utilized to get the desired outcomes on two occasions, i.e., hybrid fluid flow and micropolar fluid flow. The major findings of the current study are reported below:

- A larger pressure gradient minimizes fluid velocity.
- A larger inertia parameter minimizes the rotation profile in the case of micropolar fluid flow but ameliorates the same in the case of hybrid nanofluid flow.
- Escalation in Brinkmann number causes the amelioration in the fluid temperature.
- Magnetic field and radiation parameters mitigate the fluid temperature.
- $Bi_{2}$,$Br$,${\text{a}}_{j}$, $Ra$ and $Gr$ are escalating the entropy generation.
- $Gr$ Ameliorates the Bejan number near the midpoint of the channel and minimizes the same at other areas.

## Data Availability

The generated data during the current study available from the corresponding author on reasonable request.
